# Iodide Removal by Resorcinol-Formaldehyde Carbon Aerogels

**DOI:** 10.3390/ma15196885

**Published:** 2022-10-04

**Authors:** Andrea Domán, Bekassyl Battalgazy, Gábor Dobos, Gábor Kiss, Zhandos Tauanov, Krisztina László, Antonis A. Zorpas, Vassilis J. Inglezakis

**Affiliations:** 1 Surface Chemistry Group, Department of Physical Chemistry and Materials Science, Faculty of Chemical Technology and Biotechnology, Budapest University of Technology and Economics, Műegyetem rkp. 3, 1111 Budapest, Hungary; 2 Environmental Science & Technology Group (ESTg), Department of Chemical & Materials Engineering, School of Engineering, Nazarbayev University, Qabanbay Batyr Ave 53, Nur-Sultan 010000, Kazakhstan; 3 Surface Physics Laboratory, Department of Atomic Physics, Budapest University of Technology and Economics, Műegyetem rkp. 3, 1111 Budapest, Hungary; 4 Faculty of Chemistry and Chemical Technology, al-Farabi Kazakh National University, 71 al-Farabi Ave., Almaty 050040, Kazakhstan; 5 Laboratory of Chemical Engineering and Engineering Sustainability, Faculty of Pure and Applied Science, Open University of Cyprus, Giannou Kranidioti 33, Latsia, Nicosia 2220, Cyprus; 6 Chemical and Process Engineering Department, University of Strathclyde, 75 Montrose Street, Glasgow G1 1XJ, UK

**Keywords:** carbon aerogels, resorcinol–formaldehyde, iodide, adsorption

## Abstract

The adsorption technique is widely used in water purification, and its efficiency can be significantly improved by target-specific adsorbent design. Research on iodine and its ion removal from water has attracted a great deal of interest due to increased concentrations in the environment and acute toxic effects, e.g., in human thyroid cells. In this work, the iodide removal performance of two high-surface-area resorcinol–formaldehyde-based carbon aerogels was studied under acidic conditions. The BET surface area was 790 m^2^/g (RF_ac) and 375 m^2^/g (RMF-GO), with a corresponding micropore ratio of 36 and 26%, respectively. Both aerogels showed outstanding adsorption capacity, exceeding the reported performance of other carbons and Ag-doped materials. Owing to its basic nature, the RMF-GO carbon aerogel showed higher I^−^ capacity, up to 97 mg/g, than the acidic RF_ac, which reached a capacity of 82 mg/g. The surface chemistry of the aerogels also played a distinct role in the removal. In terms of kinetics, RF_ac removed 60% of the iodide ions and RMF-GO 30% within 8 h. The removal kinetics was of the first order, with a half-life of 1.94 and 1.70 h, respectively.

## 1. Introduction

Water treatment is becoming of greater importance year after year due to the rapid decrease in the Earth’s drinking water supply and the high degree of water pollution caused by human activity [1]. Iodide is a naturally occurring compound in natural waters and thus in drinking water [2]. The main anthropogenic sources of iodide are hospital wastewater (iodine is used as a contrast material in different diagnostic techniques, such as X-ray imaging) [3], agricultural usage of pesticides, fertilizers and biocides [3] and nuclear accidents [4]. Iodine radioisotopes are produced during plutonium and uranium fission reactions. For their hazardous radiological effects, they were identified as one of the most dangerous substances in the case of accidental release [4,5,6,7]. Increased radioiodine traces were detected in drinking water, seawater, and surface water after the Fukushima nuclear power plant accident in Japan [8,9,10,11,12]. In addition to the harm that iodide causes to the ecosystem, it has a large impact on human health and is capable of accumulating in the human body. One possible cause is the disinfection of drinking water, which leads to the formation of toxic iodinated by-products (I-DBPs) [3,13]. Excess iodide in the human body may lead to the dysfunction of thyroids due to its effect on thyroxine production, which has a direct impact on the human body’s metabolic activity [14].

Adsorption is widely used in water purification in combination with other technologies, such as flocculation, coagulation, biological oxidation, sedimentation, oxidation/degradation processes and membrane processes [15,16,17,18]. Porous materials, including carbons, have been extensively used in water treatment [19,20,21,22]. For instance, zeolites which are very common materials used for water purification, are not suitable for the removal of iodide without modification as they are cation exchangers. Impregnation with silver is a common modification used in zeolites and other materials but it increases the cost of the material. Several other materials have been used for iodide removal from water, but carbons exhibit numerous advantages because of their excellent properties and high adsorption capacity [14,21]. Table 1 presents carbon materials used for the removal of iodide from aqueous solutions. A detailed review on halide removal from waters by several materials is provided by Watson et al. [19].

Carbon-based 3D structures have attracted great interest due to their outstanding properties, such as the interconnected, hierarchical pore system, huge surface area and high chemical resistance. Their versatility makes them excellent candidates for water remediation by adsorption [32,33]. The chemical combination of various carbon materials with one another and a range of different elements via strong covalent bonds leads to composite systems with improved properties. It results in exhibiting their characteristics of high density, high hardness and high strength [34,35]. Carbon aerogels are widely studied as representatives of 3D carbon materials. They can be considered as colloid dispersions with extreme porosity, more than 95%. Within their structures, carbon particles form a porous matrix and air fills the pore system as the dispersed phase [36]. They have several potential applications, such as insulation [37], catalysis and electrocatalysis [38,39], energy storage [40], environmental remediation [41], water treatment [25] and separation technologies [42]. For more than two decades, sol–gel techniques have been applied to prepare polymer aerogels with tailored structures, which are excellent precursors for carbon aerogels [43]. Since the pioneering work of Pekala, resorcinol (R) and formaldehyde (F) have been the most widely used monomers [43]. RF gels can be produced via the polycondensation of resorcinol and formaldehyde in a basic aqueous solution. Their pyrolysis may lead to carbon aerogels [44,45]. The morphology and the surface chemistry both of the polymer and the carbon gel are tunable in various ways [46]. The technique of solvent removal plays a decisive role in the formation of pore structure [47], introducing various additives, such as carbon nanoparticles [48], heavy metals [25,48] or heteroatoms [39,49], and surface modification leads to the formation of desirable mechanical, catalytic and physical chemistry properties.

Carbon aerogels have been used for iodide removal from aqueous solutions modified by silver [23,24], organic compounds [30] and in the form of graphene quantum dots [50] or in combination with electrosorption [51] and capacitive deionization technology [52]. Sanchez-Polo et al. studied the application of silver-modified resorcinol–formaldehyde carbon aerogels for the removal of iodide from drinking water [25,53]. The silver content of the modified aerogel was 4–10% *w*/*w* and the achieved capacity for iodide was very low at 3 µmol/g, but they were using a low initial iodide concentration as well, up to 10 µmol/L. Sun et al. developed a biomass carbonaceous aerogel modified with 3-glycidyl-oxypropyl-trimethoxy-silane for the removal of iodide from aqueous solutions [30]. Experiments were carried out with an initial iodide concentration between 1 and 20 mmol/g, and the maximum adsorption capacity was 2.5 mmol/g at an initial pH of 1.5. The adsorption mechanism was mainly driven by electrostatic interaction between protonated oxygen atoms on the surface and iodide ions. In addition, Sanchez-Polo et al. studied the removal of iodide from drinking water by silver-activated carbon aerogels under dynamic conditions [23]. According to the results, a decrease in absorption capacity was observed and they claimed that it might have been due to the blockage of dissolved organic matter in the water, which reduced the access of iodide to Ag-impregnated sites. Despite this fact, the adsorption value reached 5 mmol/g at a concentration of 1.5 mmol/L in a column with a height of 8 cm and inner diameter of 1 cm, while the flow rate was 1.5 mL/min [23].

In this study, we present the synthesis, characterization and application of resorcinol–formaldehyde carbon aerogels for the removal of iodide from aqueous solutions. The aim is to address the main challenge in the field, which is the scarcity of materials tailored specifically to iodide removal from water. The literature review presented above demonstrates that resorcinol–formaldehyde carbon aerogels have been used only in a few studies for the removal of iodide from aqueous solutions, and only in their silver form. Moreover, all relevant studies on carbon aerogels were performed under a neutral pH and at concentrations below 20 ppm, while, in the present study, the concentration is up to 2000 ppm, while the pH is at 2.5. These conditions are necessary to ensure the attainment of the saturation capacity of the material and avoid the potential formation of iodate (IO_3_) at high pH values.

## 2. Materials and Methods

### 2.1. Materials and Synthesis

Iodide adsorption performance was investigated on two resorcinol–formaldehyde (RF)-based carbon aerogels of different surface chemistry and porosity. For one of the samples, named RF_ac, the RF-based carbon aerogel was obtained according to Lin and Ritter [54]. First, 1.5990 g resorcinol and 0.0308 g sodium carbonate catalyst (both from Merck) were dissolved in 48 mL distilled water. Then, 2.2 mL of 37% aqueous formaldehyde was added and the pH of the solution was set to 6.0 by HNO_3_ (Merck). After thorough mixing, the solution was poured into glass vials, sealed and cured at 85 °C for 7 days. The cured hydrogel rods (4 mm × 8 cm) were immersed in acetone to exchange the water for acetone. The solvent was removed by supercritical CO_2_ (Messer) extraction [47]. The polymer aerogel was then converted to carbon with pyrolysis in an inert N_2_ atmosphere. This carbon aerogel was post-treated by cc. HNO_3_ to enhance the surface oxygen content. The acid residue was removed by extraction with water in a Soxhlet extractor until neutral pH was reached. The nitrogen and reduced-graphene-oxide-doped carbon aerogel (named RMF-GO here) was obtained by adding melamine (Merck) and graphene oxide to the aqueous precursor mixture before the polycondensation reaction in order to incorporate nitrogen heteroatoms [48]. The melamine/resorcinol and the graphene oxide/resorcinol ratios were 0.60 and 0.12, respectively. The graphene oxide was prepared by the improved Hummers’ method from natural graphite (Graphite Tyn, China) [55]. The same procedure (but acidic treatment) was followed as above.

### 2.2. Characterization

A scanning electron microscope (SEM) (ZEISS Crossbeam 540) operating in LV mode at 15 kV, equipped with a backscattered electron detector, was used for morphological characterization. Energy-dispersive X-ray spectroscopy (EDX) combined with SEM (Oxford Instruments) was used for surface element analysis before and after I^−^ adsorption by spot and area analyses. For pore structure characterization, nitrogen adsorption–desorption isotherms were recorded with the Nova 2000e (Quantachrome) instrument at −196 °C. The samples were outgassed at 110 °C for 24 h in vacuum before measurement. The apparent surface area (*S*_BET_) was calculated using the Brunauer–Emmett–Teller (BET) model [56]. The total pore volume *V*_tot_ was derived from the amount of vapor adsorbed at relative pressure *p/p_0_*→1, assuming that the pores were filled with liquid adsorbate. The micropore volume *V*_micro_ was derived from the Dubinin–Radushkevich (DR) plot [57]. The pore size distribution (PSD) was calculated using quenched solid density functional theory (QSDFT), assuming cylinder-shaped pores at RF_ac and slit/cylinder-shaped pores in RMF-GO. Transformation of the primary adsorption data and pore size analysis were performed with the Quantachrome^®^ ASiQwin software (version 3.0). Particle size distributions (PSD) of RF_ac and RMF-GO carbon aerogels were determined with the Mastersizer 3000 (Malvern Instruments), using deionized water as a dispersant, in Hydro MV mode. The average of 3 measurements was employed to calculate the size, with obscuration levels between 15 and 17%. The Mastersizer v3.0 software of the instrument was used to control and perform all measurements. The surface chemical composition before the iodide uptake measurements was determined by XPS using an XR3E2 (VG Microtech) twin anode X-ray source and a Clam2 hemispherical electron energy analyzer. The base pressure of the analysis chamber was approximately 5 × 10^−9^ mbar. The MgK_α_ radiation (1253.6 eV) was not monochromatized. After subtracting Shirley-type backgrounds, a set of mixed Gaussian–Lorentzian functions were fitted to the peaks on each spectrum using CasaXPS.

### 2.3. Iodide Adsorption

For iodide adsorption measurement, KI (Sigma Aldrich, St. Louis, MO, USA, 99%) was dissolved in Millipore water to prepare solutions with iodide concentrations between 500 and 2000 mg/L. The pH of the I^−^ solution was adjusted to 2.5 with 37% HCl. Adsorption experiments were conducted at 24 °C ± 1 °C temperature and static conditions, without agitation, in duplicate. The iodide adsorption was attained by UV–Vis spectroscopy (PhotoLab 6600, WTW) in the wavelength range of 200–500 nm. The calibration curve was constructed from the absorbance measured at the peak maximum (227 nm) corresponding to iodide. The pH of the solutions was adjusted to 2.5 by dropwise addition of HCl, and the pH was followed with a Mettler Toledo FEP20 pH meter. Electrical conductivity (EC) was investigated with a Mettler Toledo FEP30 instrument. The pH and EC were measured after iodide adsorption as well. To study the iodide adsorption of the solution containers and the stability of the iodide, control experiments were also conducted in the concentration range of 500–2000 without carbon aerogel. The results showed that iodide was not adsorbed on the walls of the containers, the control solutions were stable, and no iodide oxidation occurred during the experiments (12–27 days). The same was observed for all equilibrium solutions, which were scanned in the range of 200–500 nm, and no iodine or triiodide was detected.

#### 2.3.1. Kinetics

For the kinetics study, 0.1 g carbon aerogel was placed into a permeable sample holder (tea bag) to avoid the dispersion of the particles into the solution, which creates problems during sampling. Then, they were immersed in 20 mL of iodide solution of 500 ppm in a plastic centrifuge tube without agitation at 25 °C. The solutions were sampled (50 μL) after 0.5, 1, 1.5, 2, 3, 4, 5, 6, 7 and 8 h and then every day until equilibrium was achieved. The concentration–time plots were fitted to the following formula:

(1) $$c\left(t\right)=c_{0}e^{-kt}$$

*c*_0_ and *c*(*t*) are the initial concentration and the concentration of time *t*, respectively, and *k* is the rate constant of the uptake process. The adsorption efficiency was calculated from the difference between the initial (*c*_0_) and the actual (*c*(*t*)) I^−^ concentrations:

(2) $$\mathrm{removal}\;\mathrm{efficiency}=\frac{c_{0}-c\left(t\right)}{c_{0}}*100$$

All experiments were performed in duplicate and the average standard deviation was 11%. The total sampling volume for UV–Vis measurement was less than 5%. The sample holder showed negligible adsorption of iodide below 5%.

#### 2.3.2. Isotherms

For the determination of the I^−^ adsorption isotherms, 0.1000 g of carbon aerogel (*m*) was placed into 20 mL (*V*) I^−^ solution with different concentrations (0, 500, 650, 800, 1000, 1500, 2000 ppm). The specific adsorbed I^−^ amount ($m_{I^{-}}$) was calculated from the difference between the initial I^−^ concentration (*c*_0_) and the equilibrium (*c*_*e*_) I^−^ concentration:

(3) $$q_{e}=\frac{\left(c_{0}-c_{e}\right)V}{m}$$

All experiments were performed in duplicate and the average standard deviation was 4%. The total sampling volume for UV–Vis measurement was less than 5%.

The iodide adsorption isotherms were fitted to the Langmuir model. This model assumes the monolayer adsorption of probe species onto the energetically homogeneous surfaces and its linear form could be presented as

(4) $$\frac{c_{e}}{q_{e}}=\frac{1}{q_{m}K_{L}}+\frac{c_{e}}{q_{m}}$$

where *c_e_* (mg/L) is the equilibrium concentration of the iodide in solution, *q_e_* and *q_m_* are the equilibrium and maximum loading of iodide on the solid in mg/g, whereas *K_L_* (L/mg) is the Langmuir constant, i.e., the equilibrium constant of the adsorption. From the monolayer capacity, the surface concentration can be estimated as

(5) $$surface\;coverage=\frac{q_{m}\times N_{A}}{M_{w}\times S_{BET}}$$

*N_A_* is the Avogadro number and *M_w_* is the molar mass of the iodide ion.

## 3. Results and Discussion

### 3.1. Characterization of the Carbon Aerogels

#### 3.1.1. Morphology

**Figure 1:**
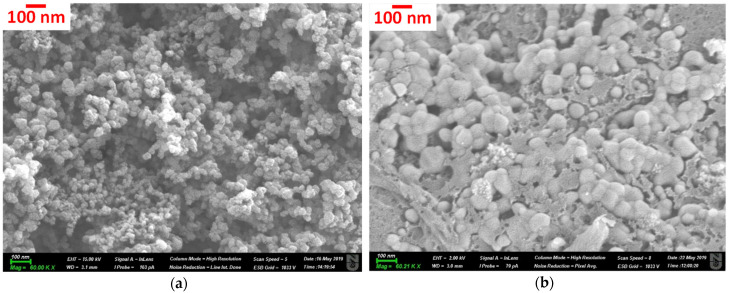
SEM images of RF_ac (**a**) and RMF-GO (**b**).

SEM images (Figure 1) show the typical loosely interconnected, complex porosity of the carbon aerogels.

**Figure 2:**
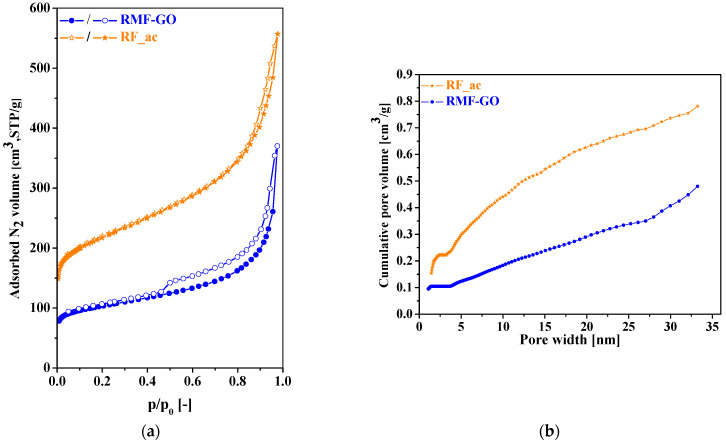
Low-temperature nitrogen adsorption/desorption isotherms (full symbols: adsorption, empty symbols: desorption branch) (**a**) and integral pore size distribution (from adsorption branch, QSDFT) (**b**) of the two carbons, RF_ac (stars, orange) and RMF-GO (dots, blue).

The difference in the mesoscopic morphological 3D structures of the two carbon materials is obvious. The melamine and graphene oxide additives result in a more compact pore structure. This is also reflected in their N_2_ adsorption/desorption isotherms (Figure 2) and the data deduced from the isotherms, shown in Table 2.

Based on the shapes of the isotherms, both carbons contain micro- and mesopores and certainly macropores. Both isotherms are of Type IVa according to the recent IUPAC report [58], revealing unrestricted monolayer–multilayer adsorption up to high *p/p_0_*. The steep run of the isotherms (also typical at isotherms of Type II) at *p/p_0_*→1 is a sign of unfilled macropores, which is also confirmed by the hysteresis loops of Type H3. This type of loop is typical of systems where the pore network consists of macropores, which are not completely filled with pore condensate. Although the quantitative detection of macropores goes beyond the limits of nitrogen adsorption, their presence is indicated by the isotherms and also supported by the SEM images.

**Figure 3:**
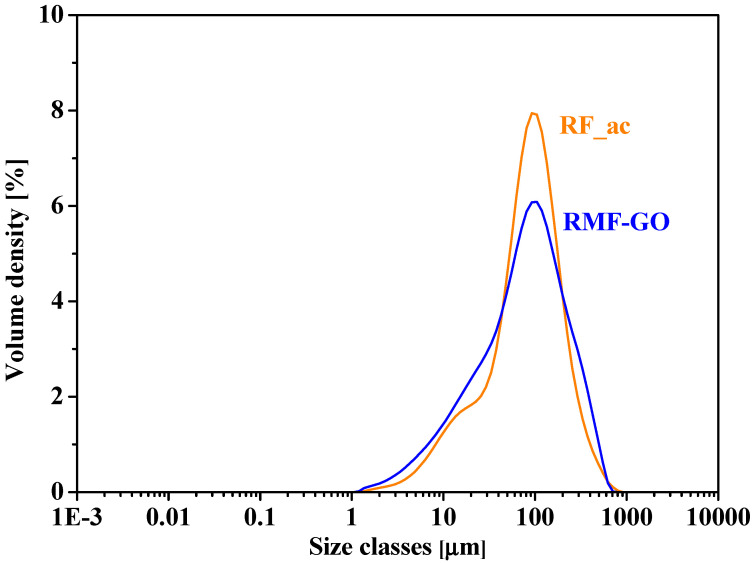
Particle size distribution of RF_ac (orange) and RMF-GO (blue) carbon aerogels.

Despite the mesoscopic and surface chemical differences, the particle size distributions of the two carbon aerogels are similar, with the most frequent size of 100 μm (Figure 3).

#### 3.1.2. Surface Chemistry

**Figure 4:**
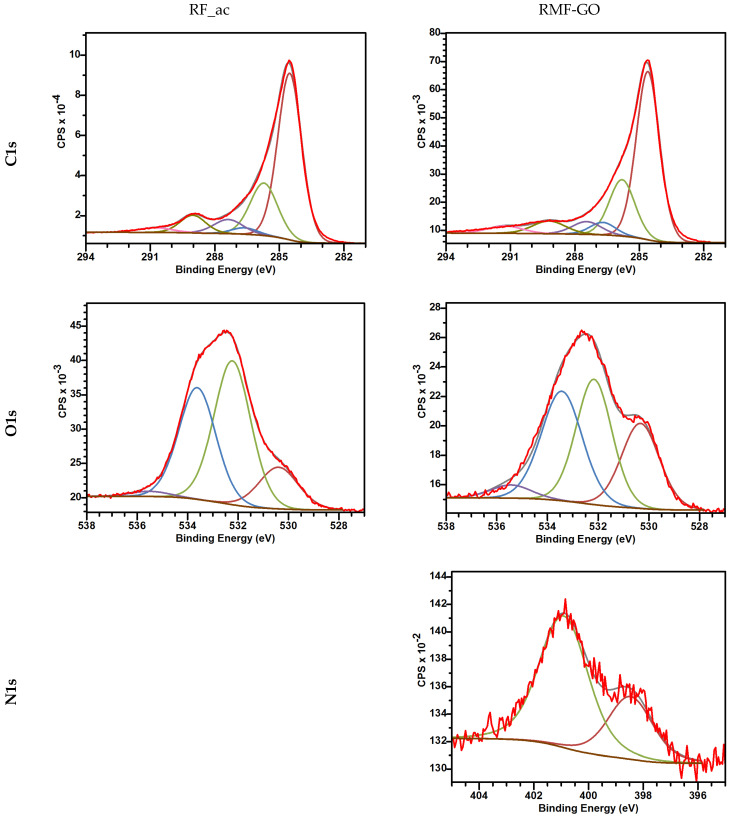
XPS spectra of RF_ac and RMF-GO carbon aerogels. Red color is the region of the spectrum corresponding to the different elements. These curves were decomposed according to the various binding states listed in Table 4 and Table 5.

The XPS results showed the difference in the surface chemical compositions of the two carbon samples (Table 3, Table 4 and Table 5 and Figure 4). The assignments in Table 4 and Table 5 were performed according to László et al. and Nagy et al., respectively [59,60]. The total heteroatom in both of them exceeds 10 at%, with a very similar distribution of the carbon species. Two forms of nitrogen were identified in the RMF-GO sample, namely N-6, i.e., pyridine and quaternary nitrogen, i.e., nitrogen substituting the carbon atom in the polyaromatic system. XPS is a vacuum technique; therefore, the surface chemistry of the RF_ac and RMF-GO carbons was also followed in aqueous solutions, similarly to the removal conditions (the pH of pure water used as a solvent was 6.08). The observed pH drop at acid-treated RF_ac (pH_RF_ac_ = 5.1) shows the dominance of acidic surface groups, while the higher pH of RMF-GO (pH_RMF-GO_ = 7.0) indicates the majority of basic groups. Most of the O-containing groups decorating the surfaces of porous carbons are acidic (carboxyl, lactone or phenolic OH), while carbonyl plus ether or pyrone groups and amino groups are basic. The graphene layer acts as a Lewis base when contacted with water [61].

### 3.2. Iodide Adsorption

#### 3.2.1. Experimental Results

The kinetics of the iodide uptake was followed at 25 °C in a 500 ppm solution. From the excellent fit to Equation (1), we could conclude that it was a first-order process on both carbons (Table 6).

**Figure 5:**
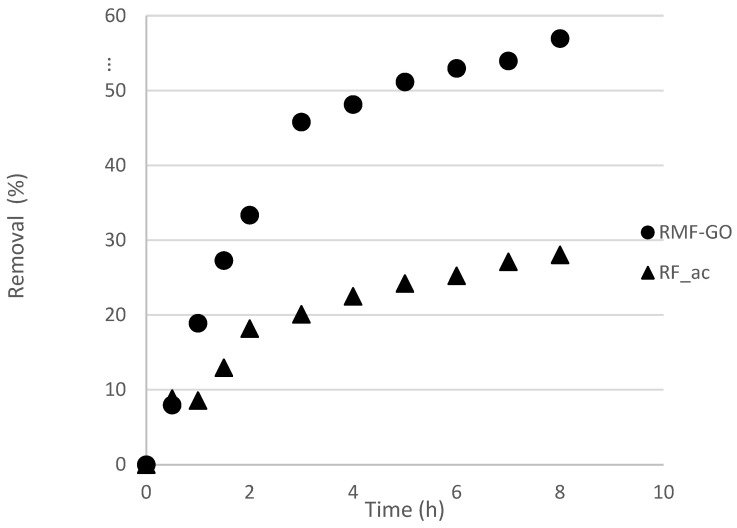
Kinetic results at an initial iodide concentration of 500 ppm, 25 °C.

**Figure 6:**
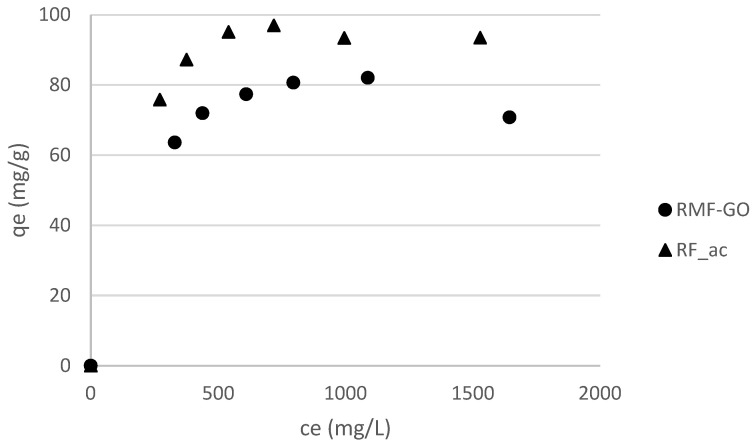
Equilibrium isotherms, 25 °C.

The particle size distributions are very similar and therefore, the diffusion within the pore structure is the governing mechanism. The kinetic results show that the adsorption on RMF-GO is faster than on RF_ac (Table 6, Figure 5). As was presumed from the textural properties (Table 2, Figure 3), the adsorption is somewhat slower in the sample with a higher micropore ratio, i.e., the average pore diameter of RF_ac is smaller, hindering the diffusion of iodide into the porous structure. Surpisingly, the lower-surface-area RMG-GO was able to remove 56% of the dissolved iodide ions, compared to 28% for the high-surface-area RF_Ac (Figure 5). The isotherms are Langmurian and the maximum capacity reaches 97 mg/g (0.76 mmol/g) for RF_ac and 82 mg/g (0.65 mmol/g) for RMF-GO (Figure 6). These values are higher than most carbons and aerogels and comparable to silver-impregnated carbons (Table 1).

The isotherms were measured in the 0–1700 ppm equilibrium concentration range at 25 °C. In the case of RMF-GO there is an outlier equilibrium point at the highest equilibrium concentration of 1644 mg/L, which leads to the decreasing trend of the isotherm, phenomenon encountered previously in literature [62].

**Figure 7:**
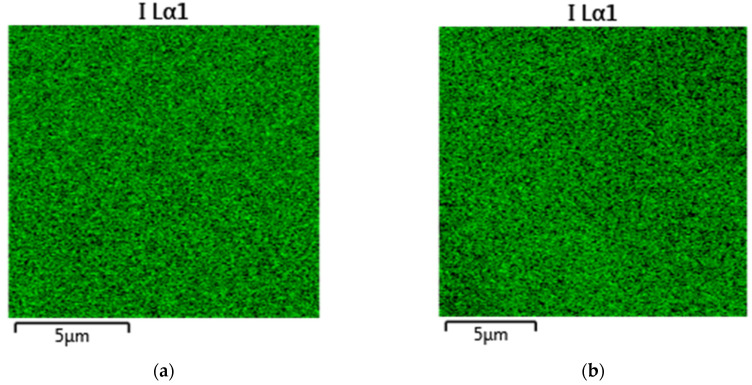
EDX mapping of RF_ac (**a**) and RMF-GO (**b**).

The SEM measurements revealed that the adsorption of iodide had no observable effect on the morphology of the surface, as expected. The EDX mapping showed that the iodide was homogeneously distributed on the surfaces of the porous carbons (Figure 7).

#### 3.2.2. Isotherms Modeling

**Figure 8:**
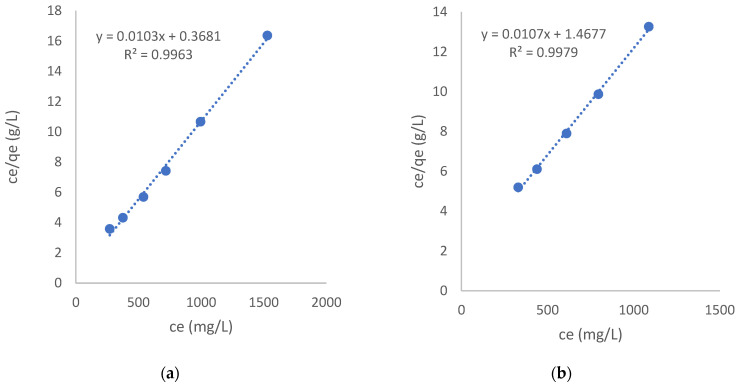
Langmuir model fit for RF_ac (**a**) and RMF-GO (**b**).

The equilibrium isotherms were fitted to the linear form of the Langmuir model in Equation (4). Due to the deviation of the isotherms at the high aqueous-phase equilibrium concentrations discussed above, the last point of the RMF-GO isotherm was excluded from modeling. From the monolayer capacity, the surface concentration can be estimated as in Equation (5). The linear plot and the derived parameters are shown in Figure 8 and Table 7, respectively. This allows us to calculate the area available for a single iodide ion.

The maximum loading derived from the model calculations is much higher than the values summarized in Table 1 from previous works (765 and 736 μmol/g for the RF_ac and the RMF-GO carbons, respectively). Both the isotherms and the fitted capacities show that the difference in the apparent surface area values is not reflected in the iodide uptake performance, due to the different surface chemistries of the carbons. The acidic carbon shows not only a weaker interaction (see the lower value of *K_L_*), but also its surface is less populated with the negative iodide ions. On the other hand, the iodide is more strongly attached to the basic surface and its surface population is much higher. Comparing the areas available for an iodide ion, it is, in both cases, significantly above the size of a hydrated iodide ion (0.34 nm^2^) [63], revealing that a great part of the surface is covered by the solvent. The substantial difference in the surface population can be attributed to the highly acidic experimental conditions, coinciding with the different surface acidic characteristics of the carbons. The low pH (2.5) was applied to ensure that the iodide was only present as I^−^. In such conditions, the surfaces of both carbons are also in protonated form. The acidic groups are therefore neutral, while the basic groups will gain a positive charge [61]. The protonated and thus positively charged nitrogen functionalities and the basic functional groups in the RMF-GO aerogel will attract the iodide anion. The Lewis base behavior of the delocalized electrons has a significant contribution to the positive surface charge in both carbons.

## 4. Conclusions

Carbon aerogels of different pore structures and surface chemistry were tested for I^−^ removal from a highly acidic aqueous medium. The first-order kinetics found was somewhat slower in the carbon with a higher micropore ratio. The surface chemistry of the aerogels played a distinct role in the removal. RF_ac removed 60% of the iodide ions within 8 h, while only 30% were removed by the other carbon during the same period. Nevertheless, the RMF-GO carbon resulted in much higher I^−^ accumulation on its surface. Due to the basic nature of this carbon at low pH conditions, the surface becomes decorated with a positive charge, attracting more iodide anions and more strongly than the acidic RF_ac. In this carbon, mainly the protonated aromatic electrons are responsible for the performance. Both carbons showed outstanding iodide binding properties from highly acidic aqueous solutions. Their performance exceeds the iodide adsorption capacity from neutral solutions of the reported Ag-doped, resorcinol–formaldehyde-based carbon species [36,37] by two orders of magnitude. Our data suggest that the adsorption properties of RF-based carbon aerogels can be easily tuned for iodide adsorption applications, without Ag doping. Future research is needed to test these materials under other conditions (e.g., neutral solutions) and to further improve the RF gels for iodine adsorption applications, by surface modification, nitrogen doping and composite formation, or by the combination of these techniques.

## Figures and Tables

**Table 1:** Carbon materials used for the removal of iodide from aqueous solutions.

Carbon Type	Iodide Concentration (mmol/L)	Dosage (g/L)	Maximum Loading (mmol/g)	Reference
Ag-doped carbon aerogels	0.15 (pH = 7)	-	5.8 × 10^−3^	[23]
Resorcinol–formaldehyde carbon aerogel impregnated with silver nanoparticles	0.1–10 × 10^−3^ (pH = 6.5–7)	1	2 × 10^−3^	[24]
Resorcinol–formaldehyde carbon aerogel impregnated with silver nanoparticles	0–10 × 10^−3^ (pH = 6.5–7)	1	2 × 10^−3^	[25]
TSPA * carbon aerogel precursor impregnated with silver chloride nanoparticles	2–14 (pH = 1–9)	1	5	[26]
Powdered activated carbon	10 × 10^−3^ (pH = 7)	0.1–0.6	0.23	[27]
Granular activated carbon and 1.05 wt% silver-impregnated granular activated carbon	8–1576 × 10^−3^ (pH = 5)	1	0.9–1	[28]
Activated carbon and bone char	18.5 Bq/mL ^129^I (pH = 7.4–9.6)	10	-	[29]
Silver- and silver-oxide-modified carbon spheres	- (pH = 1.5–2)	0.6	1.97	[14]
Biomass carbonaceous aerogel modified with KH-560 **	1–20 (pH = 1–5.4)	-	2.4	[30]
Sub-bituminous coal	0.01 (pH = 6.2)	1–20	-	[31]
Lignite	0.01 (pH = 3.9)	1–20	-	[31]

* bis(trimethoxysilylpropyl)amine; ** 3-glycidyl-oxypropyl-trimethoxy-silane.

**Table 2:** Data deduced from the low-temperature adsorption isotherms *.

Sample	*S_BET_* [m^2^/g]	*V_tot_* [cm^3^/g]	*V_micro_* [cm^3^/g]	*V_meso_* [cm^3^/g]	*V_micro_/V_tot_*	*d_avg_* [nm]
RF_ac	790	0.86	0.31	0.55	0.36	4.4
RMF-GO	375	0.57	0.15	0.42	0.26	6.1

* *S_BET_*: apparent surface area; *V_tot_*, *V_micro,_* and *V_meso_* = *V_tot_* − *V_micro_*: the total, micro- and mesopore volume, respectively; *d_avg_* = 4·*V_tot/_/S_BET_*: average pore diameter, assuming cylindrical pore geometry.

**Table 3:** Surface composition of the pristine samples from XPS.

Sample	C	O	N	O/C	N/C	(O + N)/C
	Atomic%					
RF_ac	88.3	11.3	- *	0.128		0.128
RMF-GO	94.7	3.4	1.3	0.036	0.014	0.149

* below detection limit.

**Table 4:** Distribution (%) of the carbon groups.

Peak Position, eV	285.2	286.5	287.3	288.1	289.5	291.8
RF_ac	65.3	18.2	2.0	5.3	4.5	2.9
RMF-GO	70.4	12.1	3.5	5.0	3.8	3.0

**Table 5:** Distribution (%) of the oxygen and nitrogen groups.

Peak Position, eV	O1s			N1s	
	530.9–531.2	532.8–533.0	534.2–534.5	399.2	401.7
RF_ac	5.8	60.0	34.2	-	-
RMF-GO	13.3	46.3	40.4	28.5	71.5

**Table 6:** Kinetics of the uptake.

Sample	*k,* 1/h	t_1/2_ *, h	*R^2^*
RF_ac	0.357	1.94	0.97302
RMF-GO	0.408	1.70	0.99357

* *t_1/2_* = ln2/k.

**Table 7:** Modeling results.

Sample	q_m_		K_L,_ L/mg	Surface Coverage, I^−^ ion/m^2^	Area Available, nm^2^/I^−^ ion
	mg/g	μmol/g			
**RF_ac**	97.1	765	0.028	5.83 × 10^17^	17
**RMF-GO**	93.5	736	0.073	1.18 × 10^18^	8.5

## Data Availability

Not applicable.

## References

[1] Shannon M.A., Bohn P.W., Elimelech M., Georgiadis J.G., Marĩas B.J., Mayes A.M. (2008). Science and Technology for Water Purification in the Coming Decades. Nature.

[2] Fuge R., Johnson C.C. (2015). Iodine and Human Health, the Role of Environmental Geochemistry and Diet, a Review. Appl. Geochem..

[3] MacKeown H., von Gunten U., Criquet J. (2022). Iodide Sources in the Aquatic Environment and Its Fate during Oxidative Water Treatment—A Critical Review. Water Res..

[4] Zhang X., Gu P., Zhou S., Li X., Zhang G., Dong L. (2018). Enhanced Removal of Iodide Ions by Nano Cu_2_O/Cu Modified Activated Carbon from Simulated Wastewater with Improved Countercurrent Two-Stage Adsorption. Sci. Total Environ..

[5] Buesseler K.O., Jayne S.R., Fisher N.S., Rypina I.I., Baumann H., Baumann Z., Breier C.F., Douglass E.M., George J., Macdonald A.M. (2012). Fukushima-Derived Radionuclides in the Ocean and Biota off Japan. Proc. Natl. Acad. Sci. USA.

[6] Woo T.H. (2013). Atmospheric Modeling of Radioactive Material Dispersion and Health Risk in Fukushima Daiichi Nuclear Power Plants Accident. Ann. Nucl. Energy.

[7] González-García C.M., González J.F., Román S. (2011). Removal Efficiency of Radioactive Methyl Iodide on TEDA-Impregnated Activated Carbons. Fuel Process. Technol..

[8] Awual M.R., Suzuki S., Taguchi T., Shiwaku H., Okamoto Y., Yaita T. (2014). Radioactive Cesium Removal from Nuclear Wastewater by Novel Inorganic and Conjugate Adsorbents. Chem. Eng. J..

[9] Fujiwara H. (2016). Observation of Radioactive Iodine (131I,129I) in Cropland Soil after the Fukushima Nuclear Accident. Sci. Total Environ..

[10] Kosaka K., Asami M., Kobashigawa N., Ohkubo K., Terada H., Kishida N., Akiba M. (2012). Removal of Radioactive Iodine and Cesium in Water Purification Processes after an Explosion at a Nuclear Power Plant Due to the Great East Japan Earthquake. Water Res..

[11] Kubota T., Fukutani S., Ohta T., Mahara Y. (2013). Removal of Radioactive Cesium, Strontium, and Iodine from Natural Waters Using Bentonite, Zeolite, and Activated Carbon. J. Radioanal. Nucl. Chem..

[12] Ohta T., Mahara Y., Kubota T., Fukutani S., Fujiwara K., Takamiya K., Yoshinaga H., Mizuochi H., Igarashi T. (2012). Prediction of Groundwater Contamination with 137Cs and 131I from the Fukushima Nuclear Accident in the Kanto District. J. Environ. Radioact..

[13] Dong H., Qiang Z., Richardson S.D. (2019). Formation of Iodinated Disinfection Byproducts (I-DBPs) in Drinking Water: Emerging Concerns and Current Issues. Acc. Chem. Res..

[14] Yu F., Chen Y., Wang Y., Liu C., Ma W. (2018). Enhanced Removal of Iodide from Aqueous Solution by Ozonation and Subsequent Adsorption on Ag-Ag_2_O Modified on Carbon Spheres. Appl. Surf. Sci..

[15] Singh N.B., Nagpal G., Agrawal S. (2018). Rachna Water Purification by Using Adsorbents: A Review. Environ. Technol. Innov..

[16] Tandekar S.A., Pande M.A., Shekhawat A., Fosso-Kankeu E., Pandey S., Jugade R.M. (2022). Fe(III)–Chitosan Microbeads for Adsorptive Removal of Cr(VI) and Phosphate Ions. Minerals.

[17] do Nascimento F.H., Masini J.C. (2022). Vermiculite Modified with Fe^3+^ Polyhydroxy Cations Is a Low-Cost and Highly Available Adsorbent for the Removal of Phosphate Ions. Minerals.

[18] Yaqoob A.A., Ahmad H., Parveen T., Ahmad A., Oves M., Ismail I.M.I., Qari H.A., Umar K., Mohamad Ibrahim M.N. (2020). Recent Advances in Metal Decorated Nanomaterials and Their Various Biological Applications: A Review. Front. Chem..

[19] Watson K., Farré M.J., Knight N. (2012). Strategies for the Removal of Halides from Drinking Water Sources, and Their Applicability in Disinfection by-Product Minimisation: A Critical Review. J. Environ. Manag..

[20] Yu W., Dong Q., Yu W., Qin Z., Nie X., Wan Q., Chen X. (2022). Preparation of Halloysite/Ag_2_O Nanomaterials and Their Performance for Iodide Adsorption. Minerals.

[21] Tauanov Z., Inglezakis V.J. (2019). Removal of Iodide from Water Using Silver Nanoparticles-Impregnated Synthetic Zeolites. Sci. Total Environ..

[22] Yaqoob A.A., Parveen T., Umar K., Ibrahim M.N.M. (2020). Role of Nanomaterials in the Treatment of Wastewater: A Review. Water.

[23] Sánchez-Polo M., Rivera-Utrilla J., Salhi E., von Gunten U. (2006). Removal of Bromide and Iodide Anions from Drinking Water by Silver-Activated Carbon Aerogels. J. Colloid Interface Sci..

[24] Sánchez-Polo M., Rivera-Utrilla J., Salhi E., von Gunten U. (2007). Ag-Doped Carbon Aerogels for Removing Halide Ions in Water Treatment. Water Res..

[25] Sánchez-Polo M., Rivera-Utrilla J., Méndez-Díaz J., López-Peñalver J. (2008). Metal-Doped Carbon Aerogels New Materials for Water Treatments. Ind. Eng. Chem. Res..

[26] Zhang H., Hu Y., Ye X., Liu H., Li Q., Guo M., Wu Z. (2013). Iodide Adsorption from Aqueous Solutions by Bis(Trimethoxysilylpropyl)Amine Polycondensate/Silver Chloride Composites. Desalination Water Treat..

[27] Ikari M., Matsui Y., Suzuki Y., Matsushita T., Shirasaki N. (2015). Removal of Iodide from Water by Chlorination and Subsequent Adsorption on Powdered Activated Carbon. Water Res..

[28] Hoskins J.S., Karanfil T., Serkiz S.M. (2002). Removal and Sequestration of Iodide Using Silver-Impregnated Activated Carbon. Environ. Sci. Technol..

[29] Li D., Kaplan D.I., Knox A.S., Crapse K.P., Diprete D.P. (2014). Aqueous 99Tc, 129I and 137Cs Removal from Contaminated Groundwater and Sediments Using Highly Effective Low-Cost Sorbents. J. Environ. Radioact..

[30] Sun L., Zhang Y., Ye X., Liu H., Zhang H., Wu A., Wu Z. (2017). Removal of I- from Aqueous Solutions Using a Biomass Carbonaceous Aerogel Modified with KH-560. ACS Sustain. Chem. Eng..

[31] Balsley S.D., Brady P.V., Krumhansl J.L., Anderson H.L. (1998). Anion Scavengers for Low-Level Radioactive Waste Repository Backfills. Soil Sediment Contam..

[32] Chen B., Ma Q., Tan C., Lim T.T., Huang L., Zhang H. (2015). Carbon-Based Sorbents with Three-Dimensional Architectures for Water Remediation. Small.

[33] Ma Q., Yu Y., Sindoro M., Fane A.G., Wang R., Zhang H. (2017). Carbon-Based Functional Materials Derived from Waste for Water Remediation and Energy Storage. Adv. Mater..

[34] Dos Santos M.C., Maynart M.C., Aveiro L.R., da Paz E.C., dos Santos Pinheiro V. (2017). Carbon-Based Materials: Recent Advances, Challenges, and Perspectives. Reference Module in Materials Science and Materials Engineering.

[35] Jawaid M., Ahmad A., Ismail N., Rafatullah M. (2021). Green Energy and Technology Environmental Remediation Through Carbon Based Nano Composites.

[36] Zuo L., Zhang Y., Zhang L., Miao Y.E., Fan W., Liu T. (2015). Polymer/Carbon-Based Hybrid Aerogels: Preparation, Properties and Applications. Materials.

[37] Hu L., He R., Lei H., Fang D. (2019). Carbon Aerogel for Insulation Applications: A Review. Int. J. Thermophys..

[38] Nagy B., Ábrahám D., Dobos G., Madarász J., Onyestyák G., Sáfrán G., Geissler E., László K. (2014). Molybdenum Doped Carbon Aerogels with Catalytic Potential. Carbon.

[39] Seredych M., László K., Rodríguez-Castellón E., Bandosz T.J. (2016). S-Doped Carbon Aerogels/GO Composites as Oxygen Reduction Catalysts. J. Energy Chem..

[40] Biener J., Stadermann M., Suss M., Worsley M.A., Biener M.M., Rose K.A., Baumann T.F. (2011). Advanced Carbon Aerogels for Energy Applications. Energy Environ. Sci..

[41] Maleki H. (2016). Recent Advances in Aerogels for Environmental Remediation Applications: A Review. Chem. Eng. J..

[42] Smirnova I., Gurikov P. (2018). Aerogel Production: Current Status, Research Directions, and Future Opportunities. J. Supercrit. Fluids.

[43] Pekala R.W. (1989). Organic Aerogels from the Polycondensation of Resorcinol with Formaldehyde. J. Mater. Sci..

[44] Fathy N.A., Rizk M.S., Awad R.M.S. (2016). Pore Structure and Adsorption Properties of Carbon Xerogels Derived from Carbonization of Tannic Acid-Resorcinol-Formaldehyde Resin. J. Anal. Appl. Pyrolysis.

[45] Li F., Xie L., Sun G., Kong Q., Su F., Cao Y., Wei J., Ahmad A., Guo X., Chen C.M. (2019). Resorcinol-Formaldehyde Based Carbon Aerogel: Preparation, Structure and Applications in Energy Storage Devices. Microporous Mesoporous Mater..

[46] Elkhatat A.M., Al-Muhtaseb S.A. (2011). Advances in Tailoring Resorcinol-Formaldehyde Organic and Carbon Gels. Adv. Mater..

[47] Czakkel O., Marthi K., Geissler E., László K. (2005). Influence of Drying on the Morphology of Resorcinol-Formaldehyde-Based Carbon Gels. Microporous Mesoporous Mater..

[48] Nagy B., Domán A., Menyhárd A., László K. (2018). Influence of Graphene Oxide Incorporation on Resorcinol-Formaldehyde Polymer and Carbon Aerogels. Period. Polytech. Chem. Eng..

[49] White R.J., Yoshizawa N., Titirici M. (2011). Sustainable Synthesis of Nitrogen-Doped Carbon Aerogels. Green Chem..

[50] Wang C.C., Lu S.Y. (2015). Carbon Black-Derived Graphene Quantum Dots Composited with Carbon Aerogel as a Highly Efficient and Stable Reduction Catalyst for the Iodide/Tri-Iodide Couple. Nanoscale.

[51] Ying T.Y., Yang K.L., Yiacoumi S., Tsouris C. (2002). Electrosorption of Ions from Aqueous Solutions by Nanostructured Carbon Aerogel. J. Colloid Interface Sci..

[52] Xu P., Drewes J.E., Heil D., Wang G. (2008). Treatment of Brackish Produced Water Using Carbon Aerogel-Based Capacitive Deionization Technology. Water Res..

[53] Sánchez-Polo M., Rivera-Utrilla J., von Gunten U. (2007). Bromide and Iodide Removal from Waters under Dynamic Conditions by Ag-Doped Aerogels. J. Colloid Interface Sci..

[54] Lin C., Ritter J.A. (1997). Effect of Synthesis PH on the Structure of Carbon Xerogels. Carbon.

[55] Marcano D.C., Kosynkin D.V., Berlin J.M., Sinitskii A., Sun Z., Slesarev A., Alemany L.B., Lu W., Tour J.M. (2010). Improved Synthesis of Graphene Oxide. ACS Nano.

[56] Brunauer S., Emmett P.H., Teller E. (1938). Adsorption of Gases in Multimolecular Layers. J. Am. Chem. Soc..

[57] Dubinin M.M., Radushkevich L.V. (1947). The Equation of the Characteristic Curve of Activated Charcoal. Proc. Acad. Sci. USSR Phys. Chem. Sect..

[58] Thommes M., Kaneko K., Neimark A.V., Olivier J.P., Rodriguez-Reinoso F., Rouquerol J., Sing K.S.W. (2015). Physisorption of Gases, with Special Reference to the Evaluation of Surface Area and Pore Size Distribution (IUPAC Technical Report). Pure Appl. Chem..

[59] László K., Tombácz E., Josepovits K. (2001). Effect of Activation on the Surface Chemistry of Carbons from Polymer Precursors. Carbon.

[60] Nagy B., Villar-Rodil S., Tascón J.M.D., Bakos I., László K. (2016). Nitrogen Doped Mesoporous Carbon Aerogels and Implications for Electrocatalytic Oxygen Reduction Reactions. Microporous Mesoporous Mater..

[61] Radovic L.R., Scharz J.A., Contescu C.I. (1999). Chemistry of Activated Carbon Materials. Surfaces of Nanoparticles and Porous Materials.

[62] Everett D.H. (1986). Reporting Data on Adsorption from Solution at the Solid/Solution Interface (Recommendations 1986). Pure Appl. Chem..

[63] Nightingale E.R. (1959). Phenomenological Theory of Ion Solvation. Effective Radii of Hydrated Ions. J. Phys. Chem..

